# The Temporal Pattern of Mating Behavior of the Fruit Fly, *Anastrepha zenildae* in the Laboratory

**DOI:** 10.1673/031.011.15101

**Published:** 2011-11-09

**Authors:** Lucia M. de Almeida, Arrilton Araújo, Norma H.D. Mendes, João M.G.A. de Souza, Alexandre A.L. Menezes

**Affiliations:** ^1^ Laboratório de Cronobiologia, Departamento de Fisiologia; Universidade Federal do Rio Grande do Norte; ^2^ Laboratório de Biologia Comportamental, PPG em Psicobiologia, Universidade Federal do Rio Grande do Norte; ^3^ Laboratório de Biologia Evolutiva de Insetos, Departamento de Biologia Celular e Genética, Universidade Federal do Rio Grande do Norte

**Keywords:** copulation, courtship, reproductive isolation, temporal isolation

## Abstract

The state of Rio Grande do Norte is an important fruit-producing and exporting area in northeastern Brazil. The success of this industry depends on fruit fly population control, especially in fly-free exporting zones. However, many fruits are not exported because of quarantine restrictions imposed by importing countries. A survey in the state has detected a considerable increase of the fruit fly, *Anastrepha zenildae* Zucchi (Diptera: Tephritidae), probably a result of the introduction of irrigated guava orchards that make fruit available all year. Knowledge of the sexual behavior of Tephritidae has great importance to pest control programs, particularly those that employ the Sterile Insect Technique. In order to characterize the reproductive behavior of *A. zenildae*, 32 individuals (16 males; 16 females) in each of six generations were submitted to an artificial 12:12 L:D cycle (750: < 1 lux, lights on 07:00–19:00) and observed over their lifetimes. The courtship and copulation occurred in leks and the episodes varied with the time of day, courtship being most frequent between Zeitgeber time (ZT) 3 and ZT 7, peaking at ZT 5–6. Copulations occurred between ZT 2 and ZT 8, with a higher frequency between ZT 5–7 and a peak at ZT 6. Mean duration was 0.28 ± 0.03 min/male (range: 5–163 min). Males in the leks attempted to copulate mainly between ZT 3 and ZT 7 with a peak at ZT 6, and males outside leks peaked at ZT 7. The different timing of sexual behaviors among related sympatric species, including *A. zenildae*, may contribute to species isolation.

## Introduction

Northeastern Brazil has recently joined world market as a major exporter of tropical fruits, and Rio Grande do Norte has a prominent position in this arena due to increased irrigation of semi-arid areas. Therefore, the need for pest control methods have also increased and the knowledge of reproductive behavior of tephritid fruit flies is relevant to improve monitoring, control, and eradication techniques.

One of the economically important species present is the fruit fly, *Anastrepha zenildae* Zucchi (Diptera: Tephritidae), a widely distributed generalist commonly attacking guava, *Psidium guajava.* The large numbers of *A. zenildae* and of the closely related and morphologically similar *A. sororcula* in guavas is probably due to the introduction of irrigated orchards that produce fruits year round (Moura and Moura 2006; Araújo et al. 2005; Araújo and Zucchi 2003; Zucchi 2000; Cannal et al. 1998; Araújo et al. 1996). Among *Anastrepha* species it is common to find sympatric species that use the same hosts (Zucchi 1988). In these instances, species isolation is presumably maintained by poorly understood isolating mechanisms, one of which might be differences in the diel patterning of sexual behaviors.

*Anastrepha* species exhibit species—specific temporal patterns of courtship and mating, possibly because of allochronic speciation or through divergence of a pre—copulatory isolating mechanism (Henning and Matioli 2006; Aluja et al. 2000, 2002; Selivon and Morgante 1997; Malavasi et al. 1983; Morgante et al. 1993). In *Bactrocera*, another species of economic interest, it has been suggested that circadian mating activity rhythms may cause allochronic species isolation (An et al. 2002, 2004; Miyatake et al. 2002).

Knowledge of the temporal aspects of reproduction is a tool to help develop monitoring programs of sympatric and morphologically similar species, and to optimally apply control techniques such as the release of sterile males. This study describes the courtship and mating behaviors of *A. zenildae.*

## Materials and Methods

### Subjects and maintenance conditions

*Anastrepha zenildae* specimens were obtained from infested juá fruits, *Zizipus joazeiro* Martius (Rosales: Rhamnaceae) collected in the semi-arid region in the Rio Grande do Norte, Brazil (5° 11′ 16″ S, 37° 20′ 38″ W). The fruits were put in plastic trays with a vermiculite layer that was sorted once a week to collect the pupae. After adult emergence individuals were identified, separated by sex, and transferred to plastic cages (30 × 30 × 30 cm). The cages were kept in a room with controlled temperature (26 ± 2 °C), 12:12 L:D cycle (750: < 1 lux, lights on from 07:00 to 19:00), and were supplied with water and an artificial diet (protein hydrolysate, Sustagen (www.sustagen.com.au), bee honey, unrefined sugar, yeast extract, and refined sugar) *ad libitum.*

The papaya, *Carica papaya L.* (Brassicales: Caricaceae) was used as a substrate for oviposition and larval development of females (generation F1 to F5). The papaya was then placed in a sterile vermiculite and sieved at intervals of seven days for the collection of pupae; individuals were counted and separated into lots labeled with the date collected so that each batch of pupae corresponded to individuals of the same age.

The first generation born in the laboratory was the parental population. This population was conditioned in several rearing cages (subpopulations); all were maintained with the same diet and environmental conditions. Each following generation was formed by the mating individuals from the various subpopulations chosen at random. Males and females for the experiments were randomly chosen among sub—populations and taken to the testing room and kept in cages; diet and environmental conditions were similar to the rearing cages/room.

### Behavioral observations

Behavioral observations were made over six generations (parental to F5). Sixteen sexually mature adults (14^th^ – 16^th^ day after hatching) of each sex in all generations were randomly selected, totaling 96 males and 96 females, and were observed in plastic cages (30 × 30 × 30 cm) under the light, temperature, and diet condition described above conditions.

The observations occurred during the photophase (07:00 — ZT0 lights on time) and scotophase (19:00 — ZT12 lights off time) on three consecutive days for each generation (36 hours/generation) for a total of 432 hours of behavior recording. During the photophase, flies remained under 750 lux lighting intensity and scotophase under red light with < 1 lux intensity.

Courtship and mating behaviors were recorded on a check sheet and video camera using the “all occurrences” technique (Martin and Bateson 1994). The duration, time of occurrence, and frequency of each behavior was registered. The following behaviors were recorded: pheromone emission by male, characterized by the protrusion of the anal membranes (Sivinski and Burk 1989); lek formation, where three or more males were grouped and displayed according to the definition of Aluja and Brike (1983); copulation attempt, when the male mounted the female with no aedeagus insertion into the female's ovipositor; copulation, when the male inserted the aedeagus into the female's ovipositor (Sugayama and Malavasi 2000).

### Data analysis

Recorded behaviors were compared considering the time of day as a factor. The Kolmogorov-Smirnov test was performed for normality, and results indicated that the distribution of the data did not follow a normal distribution (*p* < 0.01) (Howell 1992). So, Friedman's Anova by rank (x*2_r_*) and Wilcoxon matched pair tests were applied using the software Statistica® 7.1 (www.statsoft.com).

## Results

The sexual behavior of *A. zenildae* comprised two phases: the courtship (calling behavior) and copulation. Male calling behavior was characterized by the wings vibrating orthogonally to the body axis, followed by pheromone emission via expansion of the lateral portions of the abdomen and exertion of the anal membranes. Males moved rapidly in circles and vibrated their wings. These vibrations produced sounds and probably dispersed the pheromone. At this stage the male usually touched the substrate with his protruded anal region. Typically the male would start calling and afterwards other males would group around the first one, forming a common display area (lek). The lek was always composed of 6.0 ± 1.0 males (n = 651 leks, all generations).

**Figure 1.**
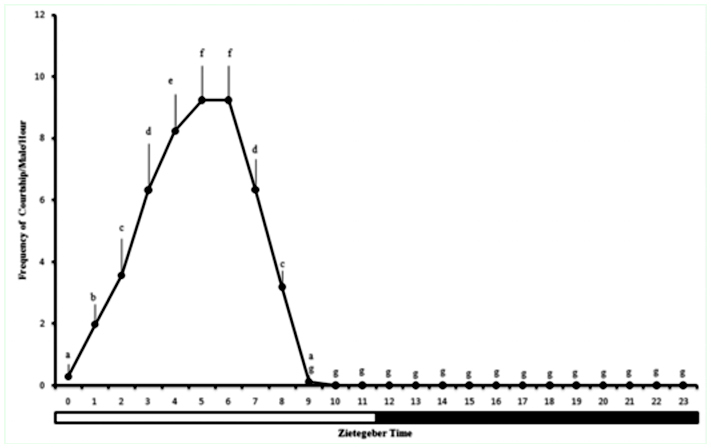
Frequency (mean ± SD) of courtship episodes of *Anastrepha zenildae* males/cage. (ZT0 = lights on time, ZT12 = lights off time). Columns with the same letters are statistically similar by Wilcoxon's test. High quality figures are available online.

**Figure 2.**
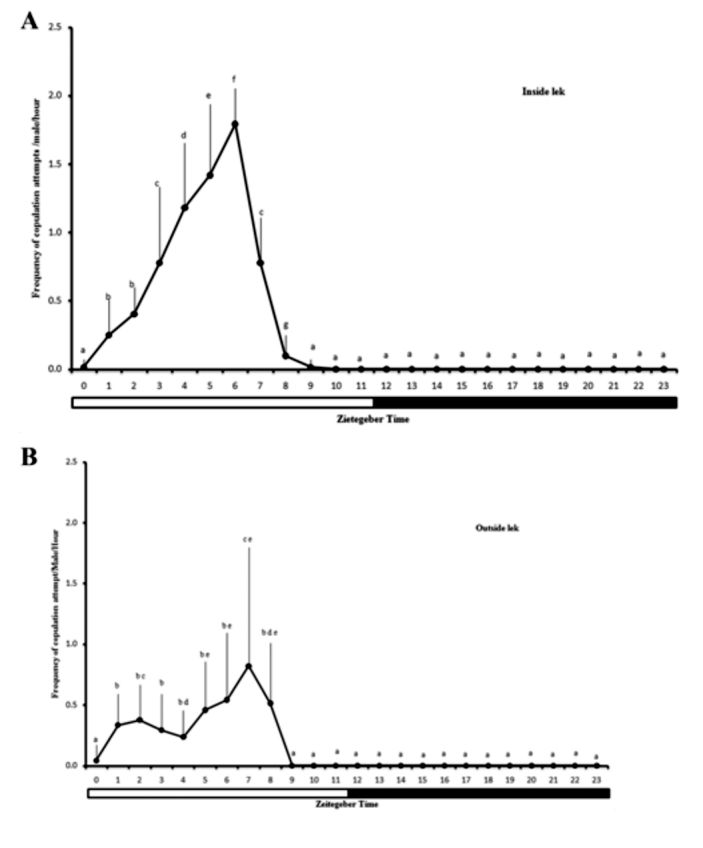
Frequency (mean ± SD) of copulation attempts of *Anastrepha zenildae* males/cage (A) inside or (B) outside the lek. (ZT0 = lights on time, ZT12 = lights off time). Columns with the same letters are statistically similar by Wilcoxon's test. High quality figures are available online.

The courtship behavior varied according to the time of the day (x*2_r_* = 400.4; *p* < 0.01); episodes occurred most frequently between ZT2 and ZT8, and peaked between ZT4 and ZT5 (Figure 1).

Copulation attempts by males were observed during the courtship inside or outside of the leks. A mean of 2.2 copulations attempts/male/hour inside the leks were recorded, with highest frequency between ZT3 and ZT7 (88.4% of the attempts), and a peak at ZT6 (x*2_r_* = 371.7; *p* < 0.01) (Figure 2A). A mean of 1.2 copulations attempts/male/hour were recorded outside the leks, with a peak at ZT 7 (x*2_r_* = 273.7; *p* < 0.01) (Figure 2B).

**Figure 3.**
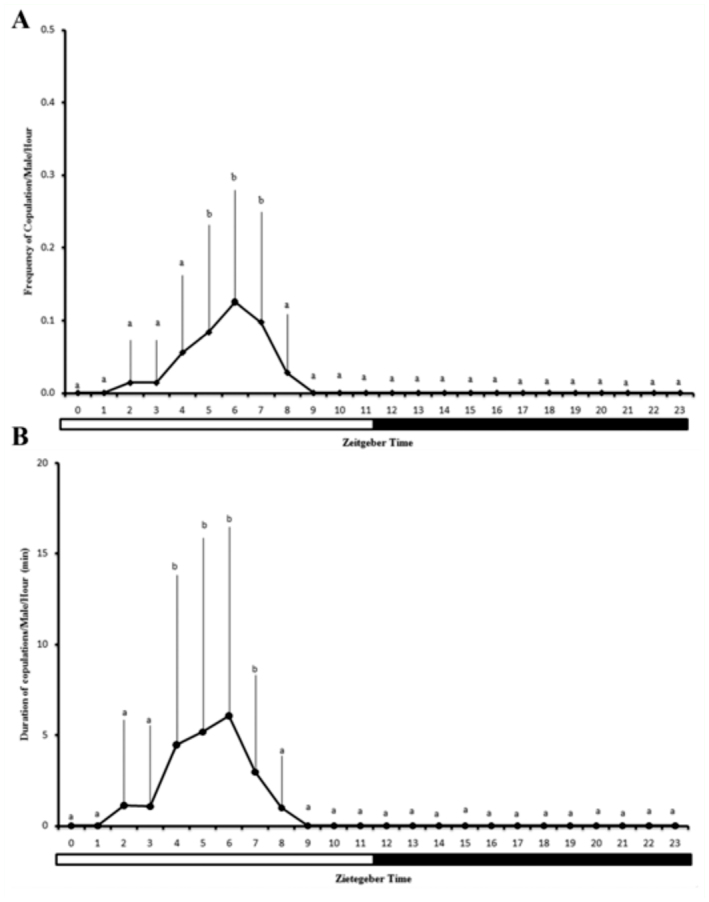
(A) Frequency of copulation and (B) duration of copulation (mean ± SD) of *Anastrepha zenildae* copulations/cage. (ZT0 = lights on time, ZT12 = lights off time). Columns with the same letters are statistically similar by Wilcoxon's test. High quality figures are available online.

Effective copulation (genital coupling) was observed from ZT2 to ZT8. This behavior had its highest frequency between ZT 5 and ZT 7, with a peak at ZT 6 (30%) (x*2_r_* =109.5; *p* < 0.01) (Figure 3A). The mean duration of copulation was 0.28 ± 0.03 min (mean ± SE, range 5.0–1630.0 min), and the longest couplings occurred between ZT4 and ZT7 (x*2_r_* =108.0; *p* < 0.01) (Figure 3B).

## Discussion

Sexually active *A. zenildae* males form groups and display courtship behavior to attract females. The group formation is typical of a lek strategy and has been observed for many species of the genus *Anastrepha*, such as *A. grandis* (Silva 1991), *A. striata* (Aluja et al. 1993; Selivon 1991), *A. pseudoparalela* (Teles da Silva et al. 1985), *A. serpentina* (Aluja et al. 1989), and various species of the *fraterculus* group to which *A. zenildae* belongs, including *A. ludens, A. obliqua* (Díaz-Fleisher et al. 2009; Aluja et al. 1983), *A. suspensa* (Dodson 1982), *A. fraterculus* (Morgante et al. 1983), and *A. sororcula* (Teles da Silva et al. 1985). In the *fraterculus* group, host ranges of sympatric species sometimes overlap.

In this study, the photoperiod started two hours after the mean time of natural sunrise. In order to compare our results with those obtained for other species under natural conditions, the sunrise was set to happen at five hours to the ZT time. Thus, ZT 0 corresponds to 05:00, which is the mean time of sunrise in our latitude (5° 11′ 16″S). Considering the corrected time, the sexual behavior of *A. zenildae* begins in the morning. The courtship episodes considerably increases as time advances, showing a peak between 10:00 and 11:00. Males inside of the leks attempted to copulate more than males outside of the leks, with a peak at 11:00. This time is coincident with the peak for effective copulations. Peak attempts by males outside leks occurred at 12:00. This implies that sexual behavior of *A. zenildae* occurs mainly before noon. This is earlier than the time recorded in other species such as *A. sororcula* and *A. obliqua*, but similar to *A. fraterculus.*

According to Oliveira and Macedo (2002), these species are sympatric with *A. zenildae* in many areas in Rio Grande do Norte. In *A. sororcula*, courtship and copulations occur from late afternoon to early evening (16:00– 19:00) (Aluja et al. 2000; Telles da Silva 1985). In *A. obliqua*, some geographical differences occur in the temporal patterns of sexual behavior. In Mexico, Aluja and Birke (1993) observed a bimodal pattern with a greater peak in the morning and a lesser one in the afternoon. López-Guillén et al. (2008) found that males of this species released volatiles all day, but the amount of volatiles was higher in the early morning. In Brazil, the courtship and copulation activities of *A. obliqua* are concentrated in the afternoon with a peak at 15:30–16:30 (Henning and Matioli 2006). In *A. fraterculus*, sexual behavior occurs in the morning (07:00–11:00) with a peak at 09:00 (Malavasi et al. 1983; Morgante et al. 1983). Copulation attempts by males outside leks were also observed in *A. suspens a* (Burk 1983; Sivinski 1989) and *A. ludens* (Robacker et al. 1991), and showed prominent peaks between 14:00 and 17:00, respectively (Burk 1983; Dodson 1982).

*Anastrepha zenildae* does not show any mating activity in the dark. Around two to three hours before ZT12 (light off), all mating behaviors ceased to happen, returning to be displayed only after ZT0 (light on) (Figure 1). According to the literature, only *A. sororcula* presents mating activity in dark (16:00–19:00) (Aluja et al. 2000; da Silva Telles 1985), similar to *Drosophila* species. Mating activity in both light and dark phases was described for seven species of *Drosophila*, and showed highly variable intensity of mating activity in the dark and dependent on the wavelength of light (Sakai et al. 1997, 2002). This effect was not observed in our data, since the light intensity and wavelength were set in light and dark times.

The results show that despite sexual behaviors being similar to other *Anastrepha* species, those of *A. zenildae* did not occur at the same time as other species in the same species group. This temporal isolation may act as a reproductive isolating mechanism among the various sympatric species. Morgante et al. (1993) and Aluja (1993) suggested that temporal isolation in *Anastrepha* might allow for sympatric speciation within the genus. In summary, the results indicate that mating activity of *A. zenildae* occurs exclusively during the light phase, acting as a reproductive isolating mechanism among sympatric species.
